# *Porphyromonas gingivalis* Induces Bisphosphonate-Related Osteonecrosis of the Femur in Mice

**DOI:** 10.3389/fcimb.2022.886411

**Published:** 2022-06-22

**Authors:** Shuxuan Wu, Feng Li, Jingjing Tan, Xiaoling Ye, Yushi Le, Nianke Liu, Vincent Everts, Qilong Wan

**Affiliations:** ^1^The State Key Laboratory Breeding Base of Basic Science of Stomatology [Hubei-Ministry of Science and Technology(MOST)] and Key Laboratory of Oral Biomedicine, Ministry of Education, School and Hospital of Stomatology, Wuhan University, Wuhan, China; ^2^Department of Stomatology, Shenzhen Yantian District People’s Hospital, Shenzhen, China; ^3^Department of Oral Cell Biology, Academic Centre for Dentistry Amsterdam, University of Amsterdam and Vrije Universiteit, Amsterdam, Netherlands; ^4^Department of Anatomy, Dental Faculty, Chulalongkorn University, Bangkok, Thailand; ^5^Department of Orthognathic & Cleft Lip and Palate Plastic Surgery, Hospital of Stomatology, Wuhan University, Wuhan, China

**Keywords:** *Porphyromonas gingivalis*, bisphosphonate-related osteonecrosis of the femur, bisphosphonate-related osteonecrosis of the jaw (BRONJ), Zoledronic acid, osteoclasts

## Abstract

One of the most prominent characteristics of bisphosphonate-related osteonecrosis of the jaw(BRONJ) is its site-specificity. Osteonecrosis tends to occur specifically in maxillofacial bones, in spite of a systemic administration of the medicine. Previous studies suggested rich blood supply and fast bone turnover might be reasons for BRONJ. Yet, a sound scientific basis explaining its occurrence is still lacking. The present study aimed to explore the role of *Porphyromonas gingivalis* (*P. gingivalis*), an important oral pathogen, on the site-specificity of bisphosphonate-induced osteonecrosis and to elucidate its underlying mechanism. Mice were intraperitoneally injected with zoledronic acid (ZA) or saline for 3 weeks. In the third week, the right mandibular first molars were extracted and circular bone defects with a diameter of 1 mm were created in right femurs. After the operation, drug administration was continued, and *P. gingivalis* suspension was applied to the oral cavities and femur defects. The mice were killed after four or eight weeks postoperatively. The right mandibles and femurs were harvested for micro-CT and histological analyses. A poor healing of bone defects of both jaws and femurs was noted in mice injected with both ZA and *P. gingivalis*. Micro-CT analysis showed a decreased bone volume, and histological staining showed an increased number of empty osteocyte lacunae, a decreased collagen regeneration, an increased inflammatory infiltration and a decreased number of osteoclasts. In addition, the left femurs were collected for isolation of osteoclast precursors (OCPs). The osteoclastogenesis potential of OCPs was analyzed *in vitro*. OCPs extracted from mice of ZA-treated groups were shown to have a lower osteoclast differentiation potential and the expression level of related genes and proteins was declined. In conclusion, we established a mouse model of bisphosphonate-related osteonecrosis of both the jaw and femur. *P. gingivalis* could inhibit the healing of femur defects under the administration of ZA. These findings suggest that *P. gingivalis* in the oral cavity might be one of the steering compounds for BRONJ to occur.

## Introduction

Bisphosphonates (BPs) are a class of chemically synthesized drugs that mainly act on bone tissue and affect bone metabolism. BPs are divided into nitrogen and non-nitrogen BPs. Zoledronate acid (ZA) is a nitrogen-containing third-generation BPs. BPs are used in a variety of bone diseases, such as osteoporosis and bone metastases of cancer. In recent years, the use of BPs in cancer patients to prevent cancer bone metastasis has been gradually increased. There are, however, unwanted side effects (Kuroshima et al., 2018a; Hayano et al., 2020). One of these is bisphosphonate-related osteonecrosis of the jaw (BRONJ). This is a detrimental effect and was first reported in 2003, where it occurred following tooth extraction in patients receiving intravenous BPs (Dannemann et al., 2007). The number of reports about BRONJ has gradually increased since then (Ruggiero et al., 2009). However, the pathophysiological mechanism of BRONJ is not yet fully understood.

Various hypotheses and theories have been put forward regarding the etiology of BRONJ, including the inhibition of BPs on bone turnover and angiogenesis, oral bacterial infection, and soft tissue toxicity (Landesberg et al., 2011; Arranz Caso et al., 2012; Hong et al., 2017; Jeong et al., 2017; Kuroshima et al., 2019; Ewald et al., 2021; Gao et al., 2021). BRONJ is likely to be multifactorial and can probably not be explained by a single cause. Interestingly, one of the most prominent characteristics of BRONJ is its site-specific effect. Osteonecrosis tends to occur specifically in maxillofacial bones, in spite of a systemic administration of the medicine (Migliorati et al., 2019). In an attempt to explain this, some hypotheses have been put forward including high bone turnover and bacterial infection in the jaw. However, a sound scientific basis explaining the site-specific effect is still lacking.

The alveolar bone of both the mandible and maxilla is a unique structure covered by a thin layer of periosteum and epithelium with an attenuated layer of connective tissue. The bone and its associated structures are subject to a variety of infections due to the presence of more than 750 bacterial species, many of which are pathogenic (Landesberg et al., 2011; Pushalkar et al., 2014). The presence of bacterial biofilms at BRONJ sites has been demonstrated by previous histomorphometric and histological studies (Sedghizadeh et al., 2008; Kaplan et al., 2009; Sedghizadeh et al., 2009). The intimate relationship of teeth and alveolar bone allows a portal of entry for microbes and other inflammatory compounds to the underlying bone; a situation that is not found in any other part of the body. In contrast to jaw bones, all bones in the body (including femurs) exist in a completely enclosed environment, without direct contact with the outside world and thus invasion of harmful microorganisms is avoided.

Previous clinical studies have shown that a large number of oral colonizing pathogens were detected at the site of osteonecrosis of patients with BRONJ (Nicolatou-Galitis et al., 2019). Recent studies highlighted an association of periodontal disease with a subsequent development or appearance of BRONJ (Tsurushima et al., 2013; Nicolatou-Galitis et al., 2015). Furthermore, some retrospective clinical studies detected a highly diverse microbial flora in necrotic bone from BRONJ patients, containing mainly anaerobic bacteria, representative of periodontal microflora. Taken together, these studies indicated the important role of periodontal pathogens in BRONJ (Hallmer et al., 2017; Ewald et al., 2021).

In the present study, we developed a bisphosphonate-induced femur osteonecrosis mouse model and investigated the relationship between BRONJ and an oral bacterial infection. We hypothesized that bone tissue, besides jaws, affected by ZA and with bacterial infection might develop osteonecrosis. We chose femurs as our research object; this bone differs from the jaw in location and type. And we chose the periodontal pathogen *Porphyromonas gingivalis* (*P. gingivalis*), as a representative of the oral flora. By comparing the occurrence of osteonecrosis of the jaws and femurs after receiving ZA-treatment, we intended to determine the possible bone-site specific relationship between osteonecrosis and *P. gingivalis* infection.

## Materials and Methods

### Bacterial Strains and Growth Condition

*P. gingivalis* (ATCC33277, VA) was cultured in trypticase soy broth (TSB) supplemented with 1% yeast extract, 5 µg/ml hemin and 1μg/ml menadione at 37°C in an anaerobic atmosphere of 80% N_2,_10% H_2_ and 10% CO_2_. *P. gingivalis* was cultured overnight, harvested by centrifugation at 6000 g for 10 min at 4°C, then washed twice with sterile phosphate buffered saline (PBS). *P. gingivalis* was resuspended in 2% carboxymethylcellulose sodium (CMC) solution. The number of bacteria (colony-forming units/ml, CFU/ml) was determined by measuring the optical density (OD) at 600 nm and extrapolating using a standard curve.

### Development of a Mouse Model of Bisphosphonate-Related Osteonecrosis of Jaw and Femur

Twenty-four female C57/BL6 mice aged 4 weeks were purchased from Center for Disease Control and Prevention, Hubei, China. Animals were housed in a specified-pathogen free facility and all procedures were performed in accordance with Institutional Animal Care and Use Committee approval of Wuhan University (S07920080B). Mice in ZA-treated groups were given ZA (125μg/kg, Sigma-Aldrich, MO) diluted in 0.9% sterile saline solution by intraperitoneal injection twice a week for 3 weeks. This dose and the drug treatment plan were based on a protocol currently used to complement oncological therapy in humans, adapted to mice (Agarwala et al., 2020). Mice in ZA-untreated groups were given 0.9% sterile saline solution in the same way.

At the third week, teeth were extracted and femur defects were created in all mice. All operations were performed under anesthesia by intraperitoneal injection of 10% pentobarbital sodium (5 ml/kg). The mandibular right first molars were extracted using vessel clamps and the integrity of the roots was checked after extraction. Defects of the right femur were made by using a trephine bur with a diameter of 1.0 mm in a high-speed dental handpiece. A round defect with a diameter of 1.0 mm in the lateral aspect of the right mid-femur was created throughout the unilateral cortical bone without destroying the opposite cortical bone. After gently washing the wound with saline, the musculature, fascia and skin were sutured in layers. After the surgery, the mice’ diet, weight and physical status were carefully monitored.

All mice were given ZA or saline as before until the end of the experimental periods. The live *P. gingivalis* solution was centrifuged and resuspended in 100μl of 2% CMC solution. For the mandibles, 100μl CMC suspension containing 1x10^8^ CFU *P. gingivalis* was applied to the extraction sockets of all mice. A week after *P. gingivalis* administration, plaque around the extraction areas was collected using a cotton swab and cultured under anaerobic conditions. For the femurs, the group of *P. gingivalis*-treated mice was injected with 100 μl CMC suspension containing 1x10^8^ CFU *P. gingivalis* into the femur defects using a 1ml insulin needle, while the group of *P. gingivalis*-untreated mice was injected with 100μl CMC solution. The needle tip penetrated the soft tissue to the bone surface around the femur defects, then the suspension was injected at an even and slow speed. The CMC solution with or without live *P. gingivalis* were applied to mice twice a week for 4 or 8 weeks.

**Figure 1:**
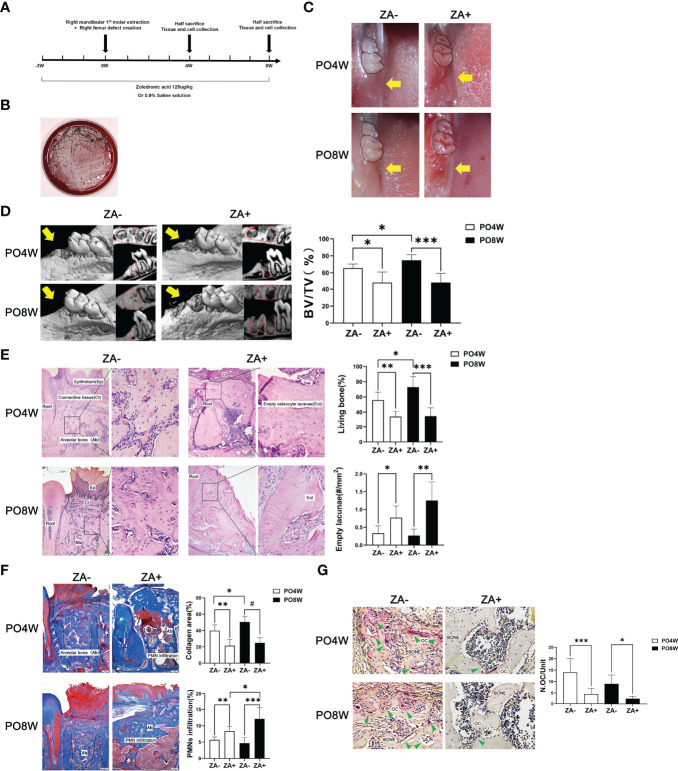
Establishment of a mouse model and impaired healing of soft and osseous tissue at tooth extraction sites under ZA administration. **(A)** Establishment of a mouse model of bisphosphonate related osteonecrosis. Schematic drawing of the procedures conducted in this study. ZA (125ug/kg) or 0.9% saline solution was administered intraperitoneally two times per week from the start of the experiment until euthanasia. At the third week, all animals received surgical intervention including right mandibular first molar extraction and the creation of right femur defects. Mice were euthanized at either four or eight weeks after surgery. Tissues and cells were collected for further analysis. **(B)** Characteristic black colonies of *P. gingivalis* were formed on a blood agar plate. **(C)** Representative images of mandibular extraction wounds. At 4 or 8 weeks postoperatively, the extraction wounds healed well (yellow arrow) in the ZA- group (M); while in the ZA+ group (M) the oral mucosa closed incompletely and open wounds (yellow arrow) were observed. **(D)** Representative images of axial and sagittal Micro-CT slices and three-dimensional views of the mandible extraction area at week 4 and 8 postoperatively. At 4 weeks postoperatively, bone healing and remodeling were detected in mandibles of the ZA- group (M); while little bone regeneration was observed in the ZA+ group (M). At 8 weeks postoperatively, in the ZA- group (M) the extraction sockets had remodeled completely and were filled with new bone; while in the ZA+ group (M) destruction and enlargement of cortical bone were detected. Quantitative evaluation is also shown. BV/TV, bone volume/tissue volume. Yellow arrows indicate the extraction sockets. **(E)** Representative sagittal H-E-stained sections and quantitative evaluation of the histological parameters of tooth extraction sockets (bar: 100μm and 20μm, 10× and 40× magnification, respectively). No necrotic bone and open wound areas were noted in the ZA- group (M); in the ZA+ group (M) clustered empty lacunae were detected around the extraction sockets. **(F)** Representative sagittal trichrome-stained sections and quantitative evaluation of the histological parameters of mandible extraction sockets (bar: 100μm, 10× magnification). There were less collagen fibers and more PMNs in the ZA+ group (M) compared to the ZA- group (M). **(G)** Representative histological TRAP-stained sections of tooth extraction sockets (bar: 20μm, 40× magnification). Statistical analysis showed that in the ZA+ group (M) the number of osteoclasts was significantly decreased. Ep, epithelium; Ct, connective tissue; Ab, alveolar bone; Eol, empty osteocyte lacunae; PMN, polymorphonuclear leukocytes; Oc, osteoclasts; PO4W, postoperative 4 weeks; PO8W, postoperative 8 weeks. *p<0.05, **p<0.01, ***p<0.001, ^#^*p*<0.0001.

Half of the number of mice were euthanized by intraperitoneal injection of excessive pentobarbital sodium at 4 and the other half at 8 weeks post-surgery. The right mandibles and femurs were examined and harvested for micro-computed tomography (micro-CT) scan and histological analysis, and the left femurs were collected for isolation of osteoclast precursors (OCPs) (**Figure 1A**).

According to ZA administration or not, the mandible (M) specimens were divided into two groups: the ZA-untreated group (ZA-) (M) and the ZA-treated group (ZA+) (M). According to ZA administration and *P. gingivalis* injection to the femur (F) defects or not, the femurs were divided into four groups: (1) ZA-untreated and *P. gingivalis*-untreated group (ZA-/*P.g*-) (F), (2) ZA-untreated and *P. gingivalis*-treated group (ZA-/*P.g*+) (F), (3) ZA-treated and *P. gingivalis*-untreated group (ZA+/*P.g*-) (F), and (4) ZA-treated and *P. gingivalis*-treated group (ZA+/*P.g*+) (F).

### Bone Morphology Analysis

The right mandibles including the tooth extraction site and the right femurs were dissected from mice of each group at the fourth and the eighth week postoperatively. The specimens were fixed in 4% paraformaldehyde buffer for 48 h, and rinsed with running water overnight to prepare for micro-CT scan.

High-resolution micro-CT imaging was acquired with a Skyscan 1172 micro-CT scanner (Bruker micro-CT, Kontich, Belgium). The samples were scanned at an energy of 88 kV and 100μA intensity with a resolution of 8.665μm pixel with a filter of 1.0 mm-thick aluminum. The images were reconstructed by using the SkyScan NRecon program. The CTvox software and the SkyScan CT-analyzer software were used to obtain 3D reconstruction images and further bone morphology analysis (Kim et al., 2017). Microarchitectural properties of the mandible and femur specimens were evaluated within a conforming volume of interest (VOI). Bone volume fraction (BV/TV) was used to evaluate bone structure.

### Hematoxylin and Eosin (H&E), Tartrate-Resistant Acid Phosphatase (TRAP) and Masson’s Trichrome Staining

The right mandibles and femurs of mice were, following fixation in 4% formaldehyde solution, decalcified in 10% ethylenediamine tetra acetic acid (EDTA) in PBS 0.1 M, pH 7.4 for 40 days. Samples were then dehydrated in a graded ethanol series, cleared in xylene, infiltrated and embedded in paraffin, and sectioned at 5 μm thickness along the sagittal plane. The sections were deparaffinized in xylene, hydrated in a graded ethanol series and stained. H&E staining was performed to observe bone healing at the extraction site of the mandibles and at the wounds of the femurs. TRAP staining was performed utilizing the leukocyte acid phosphatase kit for detecting osteoclasts. Multinucleated (≥3 nuclei) TRAP-positive cells were defined as osteoclasts and counted. Masson’s trichrome staining was carried out for comparing the differences in the area of new collagen fibers and the degree of infiltration of polymorphonuclear cells (PMN) in the extraction sockets and femur defects among different groups.

To detect bisphosphonate-related osteonecrosis-like lesions at areas of interest around the extraction sockets and femur defects, four uniformly spaced (approximately 50μm) stained sections were selected per sample and analyzed using ImageJ software (Bethesda, NIH, MD). Quantitative analysis was conducted using the following bone parameters as previous studies described (Kuroshima et al., 2018a; Hayano et al., 2020; Buranaphatthana et al., 2021): (1) the percentage of vital bone area was determined by the area in which there were morphologically normal osteocytes divided by the total bone area (%); (2) the number of empty osteocyte lacunae, defined as lacunae with no visible cell remnants, was counted per square millimeter (#/mm^2^); (3) collagen fibers quantified in the remaining connective tissue (%); (4) the proportion of PMN infiltration was calculated relative to total connective tissue area (%); and (5) osteoclast numbers were assessed and related to the bone surface (#/unit).

### Immunohistochemistry

For immunohistochemistry, sections were deparaffinized, rehydrated, subjected to antigen retrieval with gastric enzyme, blocked, and then incubated with (i) rabbit anti-RANK polyclonal antibody, (ii) rabbit anti-CTSK polyclonal antibody, (iii) rabbit anti-IL-1β polyclonal antibody, (iv) rabbit anti-IL-6 polyclonal antibody and (v) rabbit anti-TNF-α polyclonal antibody (all antibodies were from Abclonal, Wuhan, China) at 1:100 dilutions at 4°C overnight. Then, the sections were incubated with biotinylated secondary antibody for 30 min and subsequently with streptavidin conjugated with horseradish peroxidase (HRP) for 20 min. The positive area was visualized by using DAB staining solution (Maixin Biotechnology, Fuzhou, China). The sections were re-stained with hematoxylin, dehydrated, mounted and observed under the microscope (Inoue et al., 2021). As control, sections were submitted to the same procedures, except for replacing the first antibody with a non-immune rabbit antibody at the same concentration. Statistical analysis was performed by Image Pro Plus (IPP, Media Cybernetics, MD). Several regions of interest (ROI) were randomly selected around the extraction sockets and femur defect areas. The number of CTSK and RANK positive cells in each field was counted and expressed as mean ± standard deviation (SD). The area of immunolabeling of IL-1, IL-6 and TNF-α was obtained through optical density assessment using a color threshold tool of IPP. The data were expressed in percentages as mean ± SD.

### Osteoclast Culture and Quantification

Osteoclasts were generated from precursor cells obtained from left femurs of mice. The femurs were isolated carefully and thoroughly dissected from muscle and periosteum under sterile conditions. Then the bilateral metaphysis of the femurs was removed and bone marrow was flushed with a 1ml syringe with α-MEM plus 10% FBS, 100 U/ml penicillin and 100 mg/ml streptomycin. The thus obtained osteoclast precursors (OCPs) cells were seeded in T-25 flasks containing 10 ng/ml recombinant mouse macrophage colony stimulating factor (M-CSF, PeproTech, NJ). After culturing for 24h in a humidified cell incubator (37°C, 5% CO_2_), cells were collected and seeded onto plates at a concentration of 1×10^6^ cells/mL and cultured in the presence of 10 ng/mL M-CSF for 3 day. The OCPs were subsequently cultured for 4 or 6 days in the presence of 30 ng/mL M-CSF and 20 ng/mL receptor activator of nuclear factor-kappa B ligand (RANKL, PeproTech) with a medium change every other day (Maridas et al., 2018). To quantify TRAP positive multinucleated cells, cells cultured for 6 days were fixed with 4% paraformaldehyde for 10 min and stained for TRAP using the leukocyte acid phosphatase kit (Sigma Aldrich) according to the manufacturer’s instructions. Osteoclasts were identified as TRAP positive cells with no less than three nuclei and were counted manually under the microscope.

### Flow Cytometry

OCPs extracted from femur marrow were seeded in T-25 flasks and cultured in the presence of 10 ng/mL M-CSF for 1 day. Then the non-attached cells were seeded in 96-well plates and cultured in the presence of 10 ng/mL M-CSF for 3 days. The adherent cells were collected as OCPs by trypsin digestion. OCPs were centrifuged and resuspended in PBS with 1% bovine serum albumin (BSA, BioFroxx, Germany). OCPs were divided into 4 groups and incubated with (a) PBS, (b) anti-CD31, (c) anti-Ly6-C or (d) anti-CD31 + anti-Ly6-C antibodies (PeproTech) at 4°C for 30 min. The OCPs were washed two times, centrifuged and resuspended with 350μl PBS containing 1% BSA. Then the OCPs were transferred into flow detection tube and measured by flow cytometry (CytoFLEX, Beckman Coulter, CA) (Flynn et al., 2020).

### Quantification of Bone Resorption

OCPs extracted from left femurs were seeded in 96-well plates on slices of bovine cortical bone inserted into the wells. The bone slices were round sections, with a 6 mm diameter and 0.4 mm thickness. The bone slices were sterilized by autoclaving before using. The cells were cultured in medium with 30 ng/mL M-CSF and 20 ng/mL RANKL for 14 days to induce maturation of osteoclasts and their resorption of bone. On the 14th day, the medium in the plates was discarded and the bone slices were washed with PBS. The bone slices were soaked in 10% ammonia and placed on a shaker for 30 min. Then the bone slices were washed with ddH_2_O for 3 times, submerged in alum at 37°C for 10 min, and washed with ddH_2_O for 3 times. Then, the bone slices were dried and stained with Coomassie Brilliant Blue (Biosharp, Hefei, China) for 2-3s. The bone resorption lacunas were observed under the microscope and the number or area of the resorption pits were measured with IPP software.

### Quantitative Real−Time PCR

Total RNA was extracted from cells on day 4 after osteoclast differentiation using EZNA Total RNA Kit (Omega, GA). Then cDNA was synthesized from 1μg total RNA using PrimeScript™RT reagent Kit (Takara, Japan) according to the manufacturer’s protocol. Quantitative real-time polymerase chain reaction (QRT-PCR) was performed with SYBR^®^ Green Realtime PCR Master Mix (TOYOBO, Japan). Parallel triplicates were set each time. Amplified PCR products were quantified and normalized using GAPDH as a control. Relative gene expression levels were calculated using the 2^−ΔΔ^CT method (Ohori et al., 2020). The sequences of PCR primers were presented in **Table 1**.

**Table 1:** Primer sequence for Quantitative Real-Time PCR.

Genes	Primer sequence
TRAP	F:5’-gACAAgAggTTCCAggAgACC-3’ R: 5’-gggCTggggAAgTTCCAg-3’
RANK	F:5’-TgggCTTCTTCTCAgATgTCTTT-3’ R: 5’-TgCAgTTggTCCAAggTTTg-3’
CTSK	F:5’-ACAgCAggATgTgggTgTTCA-3’ R: 5’-gCCgAgAgATTTCATCCACCT-3’
MMP9	F:5’-CAAAGACCTGAAAACCTCCAA -3’ R: 5’-GGTACAAGTATGCCTCTGCCA -3’
DC-STAMP	F:5’-TgTATCggCTCATCTCCTCCAT-3’ R: 5’-gACTCCTTgggTTCCTTgCTT-3’
NFATc1	F:5’-CATgCgAgCCATCATCgA-3’ R: 5’-TgggATgTgAACTCggAAgAC-3’
IL-1β	F:5’-ggACCCATATgAgCTgAAAgCT-3’ R: 5’-TgTCgTTgCTTggTTCTCCTT-3’
IL-6	F:5’-gAgTTgTgCAATggCAATTCTg-3’ R: 5’-TggTAgCATCCATCATTTCTTTgT-3’
TNF-α	F:5’-gCCACCACgCTCTTCTgTCT-3’ R: 5’-gTCTgggCCATAgAACTgATgAg-3’
GAPDH	F:5’-TgAAgCAggCATCTgAggg-3’ R:5’-CgAAggTggAAgAgTgggAg-3’

### Western Blot

On day 6 after osteoclast differentiation cellular proteins were extracted using RIPA buffer (Beyotime, Shanghai, China) supplemented with protease and phosphatase inhibitors. The protein concentration was quantified with Bicinchoninic acid kit (Beyotime). A total of 20μg of protein from each sample was separated using sodium dodecyl sulfate polyacrylamide gel electrophoresis (SDS-PAGE) and then blotted onto a polyvinylidene fluoride (PVDF) membrane. After blocking with QuickBlock™ Blocking Buffer (Beyotime) for 15 min at room temperature, the membranes were incubated at 4°C overnight with primary antibodies at 1:1000 dilution, including cathepsin K (CTSK, Abclonal), receptor activator of nuclear factor-kappa B (RANK, Abclonal), tartrate resistant acid phosphatase (TRAP, Abcam, Cambridge, UK), nuclear factor of activated T cell cytoplasmic 1(NFATc1, Abclonal), phospho-NF-kappaB p65/NF-kappaB p65 (CST, MA), MAPK(p-p38/p38, p-JNK/JNK, p-ERK/ERK) (CST), p-AKT/AKT (CST) and GAPDH (Abclonal).

After washing the PVDF membranes for three times, they were incubated with horseradish-peroxidase (HRP)-conjugated secondary antibody for 1h at room temperature. Protein bands were visualized using enhanced chemiluminescence (ECL, Advansta, CA). The chemiluminescent signal was captured by Image Studio System (LICOR, NE). The protein was quantitatively analyzed by the ratio of the grey value between the target protein and GAPDH in the same sample.

### Statistical Analysis

All experiments were conducted in triplicate and the data of three times animal experiments were combined. Data was presented as mean ± standard deviation (SD). The mean differences between two groups were analyzed by an unpaired two-tailed Student’s *t*-test and multi groups were analyzed with one-way ANOVA (GraphPad Prism, GraphPad Software, CA). *P* values less than 0.05 were considered statistically significant.

## Results

### ZA Administration Impaired Healing of Soft and Osseous Tissue at Tooth Extraction Sites

After ZA and *P. gingivalis* administration, the oral plaque around extraction sockets was collected for anaerobic culture. The presence of *P. gingivalis* was confirmed by the formation of characteristic black colonies on the blood plate (**Figure 1B**).

At four weeks after surgery, oral examination revealed the ZA+ group (M) exhibited incomplete oral mucosal closure without open wounds, while the ZA- group (M) showed good healing of tooth extraction wounds. At eight weeks after surgery, the ZA+ group (M) showed open and inflamed wounds, while the ZA- group (M) presented complete mucosal coverage without inflammation or swelling (**Figure 1C**).

Micro-CT analysis confirmed good healing and remodeling at the fourth week postoperatively in the ZA- group (M), and the extraction sockets were filled with new bone at eight weeks postoperatively with an increased BV/TV value. In contrast herewith, in the ZA+ group (M), bone fill was hardly detected at four weeks postoperatively. At eight weeks an increased destruction of cortical bone was detected as well as little bone regeneration (**Figure 1D**). Quantitative analysis demonstrated, the BV/TV values were significantly lower in ZA+ group compared with the ZA- group at both four and eight weeks postoperatively (**Figure 1D**).

Histological staining, including H&E, TRAP and Masson trichrome-staining, was applied to evaluate the healing of soft and osseous tissue. H&E staining showed the samples of the ZA- group (M) were filled with fibrous tissue with some newly formed viable woven bone at the fourth week after surgery. And the woven bone was partly replaced by trabeculae at eight weeks after surgery, which showed as normal wound healing and bone remodeling. In contrast, the samples of the ZA+ group (M) showed a significantly decreased amount of viable bone with a reduced number of osteocytes in extraction sockets at four weeks postoperatively. The number of empty lacunae was further increased at the eighth week postoperatively (**Figure 1E**). Masson trichrome-staining showed the extraction sockets of the ZA+ group (M) had less new collagen fibers and more PMN infiltration compared with the ZA- group (M) (**Figure 1F**). TRAP staining revealed specimens of the ZA+ group (M) had a significantly decreased number of osteoclasts on bone surfaces of tooth extraction sockets compared with the ZA- group (M) at both 4 and 8 weeks postoperatively (**Figure 1G**).

### ZA Administration Plus *P. gingivalis* Injection Suppressed Soft and Osseous Tissue Healing of Femur Defects

**Figure 2:**
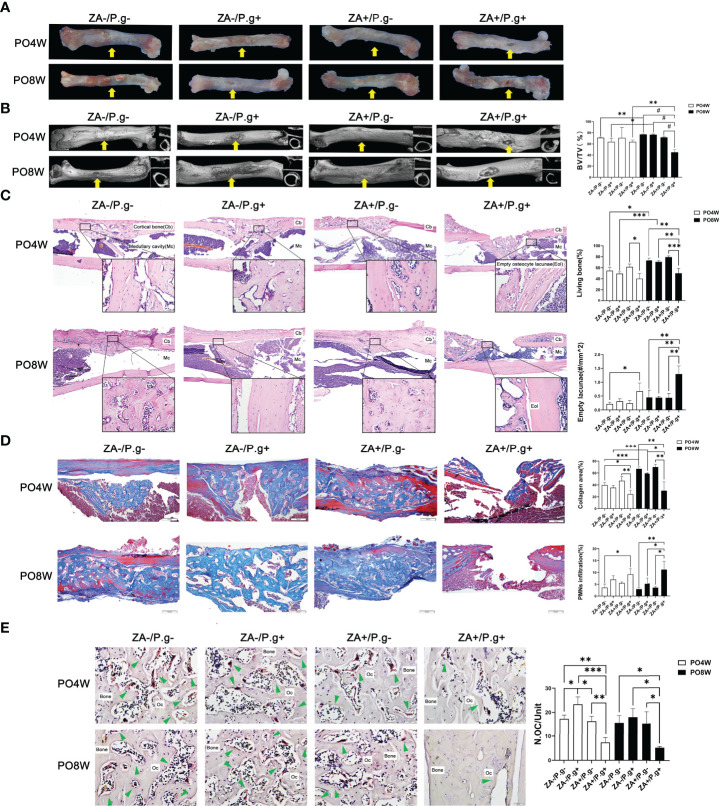
ZA administration plus *P. gingivalis* infection suppressed soft and osseous tissue healing of femur defects. **(A)** Representative images of femur defects. At 4 or 8 weeks postoperatively, in the ZA-/*P.g*-, ZA-/*P.g*+ and ZA+/*P.g*- groups (F) the bone defects healed well and bone callus was present (yellow arrow); while incomplete healing of unilateral cortical bone (yellow arrow) was observed in the ZA+/*P.g*+ group (F). **(B)** Representative axial and sagittal Micro-CT slices and three-dimensional views of femur defect areas at week 4 and 8 postoperatively. At 4 weeks postoperatively, the femur defects of the ZA-/*P.g*-, ZA-/*P.g*+ and ZA+/*P.g*- groups (F) showed callus formation and bone regeneration, while the ZA+/*P.g*+ group (F) showed poor healing of unilateral cortical bone. At 8 weeks postoperatively, the femur defects of the ZA-/*P.g*-, ZA-/*P.g*+ and ZA+/*P.g*- groups (F) showed bone regeneration and complete full thickness bridging, while the ZA+/*P.g*+ group (F) showed rare bone regeneration and poor healing. Quantitative evaluation is also shown. BV/TV, bone volume/tissue volume. Yellow arrows indicate the femur defects. **(C)** Representative sagittal H-E-stained sections and quantitative evaluation of the histological parameters of femur defects (bar: 100μm and 20μm, 10× and 40× magnification, respectively). There were unilateral incomplete healing and empty lacunae around the defect areas in the ZA+/*P.g*+ group (F). **(D)** Representative sagittal trichrome-stained sections and quantitative evaluation of the histological parameters of femur defects (bar: 100μm, 10× magnification). There were less collagen fibers and more PMNs in the ZA+/*P.g*+ group (F) compared to the other groups. **(E)** Representative histological TRAP-stained section of femur defect areas (bar: 20μm, 40× magnification). Statistical analysis showed that in the ZA+/*P.g*+ group (F) the number of osteoclasts was significantly decreased. Cb, cortical bone; Mc, Medullary cavity; Oc, osteoclasts; PO4W, postoperative 4 weeks; PO8W, postoperative 8 weeks. *p<0.05, **p<0.01, ***p<0.001, ^#^*p*<0.0001.

At four weeks after surgery, incomplete healing of cortical bone of the femur defects was noted in the ZA+/*P.g*+ group (F), while bone callus formation and complete defect healing were observed in all mice of the ZA-/*P.g*-, ZA-/*P.g*+ and ZA+/*P.g*- groups (F). At eight weeks after surgery, incomplete healing and only penetrated unilateral cortical bone of the femur defects were noted in all mice of the ZA+/*P.g*+ group (F), while complete healing and bridging were detected in the other groups (**Figure 2A**).

Micro-CT analysis revealed bone repair and new bone formation in the ZA-/*P. g*-, ZA-/*P.g*+ and ZA+/*P.g*- groups (F) at four weeks after surgery. Repair was not found in the ZA+/*P.g*+ group (F). In the former three groups callus formation and bone regeneration was apparent in the femur defects area; yet it had not completely healed. Until eight weeks postoperatively, the ZA-/*P.g*-, ZA-/*P.g*+ and ZA+/*P.g*- groups (F) showed abundant bone regeneration and complete full thickness bridging. The BV/TV values of the ZA-/*P.g*- and ZA-/*P.g*+ groups (F) were somewhat higher in the eight weeks samples compared to those of four weeks (**Figure 2B**). However, only little bone regeneration and hardly any periosteal callus was observed in the ZA+/*P.g*+ group (F) at four weeks after surgery, but there was no statistical difference in BV/TV values among groups. At the eighth week postoperatively, the ZA+/*P.g*+ group (F) showed hardly any bone regeneration with markedly decreased BV/TV values compared to the other groups (**Figure 2B**).

H&E staining demonstrated more connected bone structures in the defect area of the ZA-/*P.g*-, ZA-/*P.g*+ and ZA+/*P.g*- groups (F) at four weeks after surgery, while the ZA+/*P.g*+ group (F) had a small amount of woven bone extending into the medullary cavity. At eight weeks postoperatively, less developed cortical bone with many empty lacunae around the defects were seen in the ZA+/*P.g*+ group (F), while abundant bone regeneration and a nearly complete cortical bone bridge was apparent in the other groups (**Figure 2C**). Masson trichrome-staining supported these findings. New cortical bone bridges contained numerous new collagen fibers in the ZA-/*P.g*-, ZA-/*P.g*+ and ZA+/*P.g*- groups (F), while the ZA+/*P.g*+ group (F) showed more PMN infiltration and less collagen fiber regeneration at both time points postoperatively (**Figure 2D**). TRAP staining showed a decline in the number of osteoclasts around the defects in the ZA+/*P.g*+ group (F) compared to the other groups at both 4 and 8 weeks postoperatively (**Figure 2E**).

### ZA Administration and Local *P. gingivalis* Infection Significantly Changed the Expression Level of Osteoclastogenesis- and Inflammation-Related Genes

**Figure 3:**
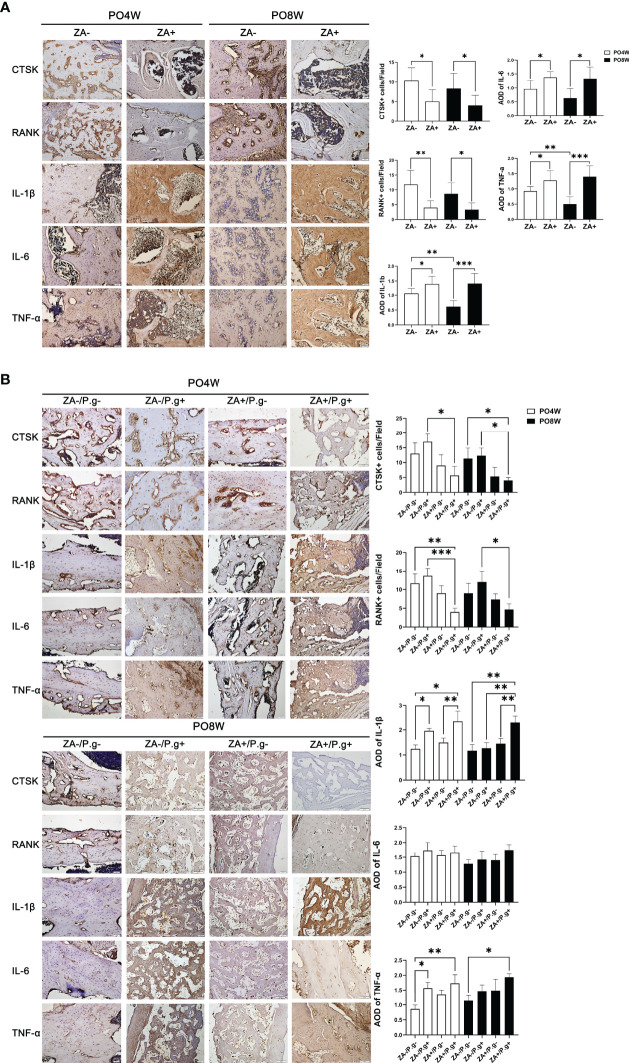
ZA administration and local *P. gingivalis* injection significantly changed the expression level of osteoclastogenesis- and inflammation-related genes. **(A)** Representative sagittal immunohistochemical staining images and statistical quantitative analysis of mandible extraction sockets (bar: 50μm, 20× magnification). The number of CTSK- and RANK-positive cells in the ZA+ group (M) were significantly lower than in the ZA- group (M) at both 4 and 8 weeks postoperatively. The immunolabeling average optical density (AOD) for IL-1β, IL-6 and TNF-α was higher in the ZA+ group (M); the degree of inflammation declined at 8 weeks compared to 4 weeks postoperatively in the ZA- group. **(B)** Representative sagittal immunohistochemical staining images and statistical quantitative analysis of femur defects (bar: 50μm, 20× magnification). Only in the ZA+/*P.g*+ group (F) a decreased number of CTSK- and RANK-positive cells was found. The immunolabeling average optical density (AOD) for IL-1 in the ZA-/*P.g*+ and ZA+/*P.g*+ groups (F) was higher than the other groups at 4 weeks postoperatively. The expression of TNF-α was also higher in the ZA-/*P.g*+ and ZA+/*P.g*+ groups (F) compared to the ZA-/*P.g*- group (F). But at 8 weeks postoperatively, the expression of these cytokines declined in the ZA-/*P.g*+ group (F) while remained high in the ZA+/*P.g*+ group (F). There was no statistical difference in the expression of IL-6 between groups. PO4W, postoperative 4 weeks; PO8W, postoperative 8 weeks. *p<0.05, **p<0.01, ***p<0.001.

Several factors involved in osteoclastogenesis (CTSK and RANK) and the inflammatory response (IL-1 etc.) were evaluated by immunohistochemical staining. As expected CTSK and RANK were expressed by osteoclasts. In mandible specimens, the number of CTSK- and RANK-positive cells in the ZA+ group (M) were significantly lower than in the ZA- group (M) at both 4 and 8 weeks postoperatively, which suggested ZA administration inhibited the formation of osteoclasts (**Figure 3A**). In femur specimens, only in the ZA+/*P.g+* group (F) a decreased number of CTSK- and RANK-positive cells was found (**Figure 3B**).

Immunolabeling of IL-1β, IL-6 and TNF-α, showed the presence of these cytokines in inflammation-related cells and the extracellular matrix. The immunolabeling optical density reflected the level of inflammation ranging from mild to moderate. In mandible specimens, the immunolabeling optical density for IL-1β, IL-6 and TNF-α was higher in the ZA+ group (M); in the ZA- group the degree of inflammation declined at 8 weeks compared to 4 weeks postoperatively (**Figure 3A**). In femur specimens, the immunolabeling optical density for IL-1 in the ZA-/*P.g*+ and ZA+/*P.g*+ groups (F) was higher than the other groups at 4 weeks postoperatively. The expression of TNF-α was also higher in the ZA-/*P.g*+ and ZA+/*P.g*+ groups (F) compared to the ZA-/*P.g*- group (F). But at 8 weeks postoperatively, the expression of these cytokines declined in the ZA-/*P.g*+ group (F) while remained high in the ZA+/*P.g*+ group (F). There was no statistical difference in the expression of IL-6 between groups (**Figure 3B**).

### Osteoclastogenesis and Bone Resorption by OCPs Extracted From Femurs Was Inhibited by ZA Administration But Not by *P. gingivalis* Infection

In order to explore the effects of ZA administration and local *P. gingivali*s injection on the formation and differentiation of OCPs, we isolated OCPs from the left femurs of mice and cultured them with M-CSF and RANKL to induce osteoclastogenesis.

**Figure 4:**
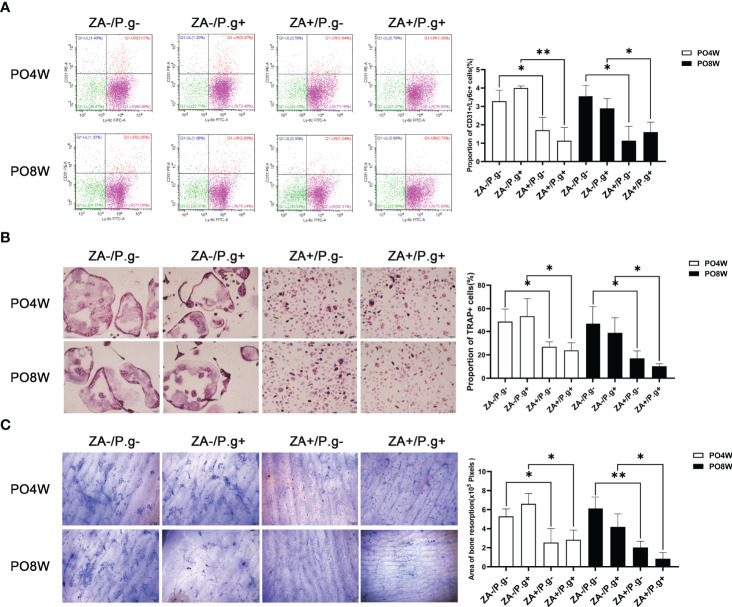
Osteoclastogenesis and bone resorption by OCPs extracted from femurs was inhibited by ZA administration but not by *P. gingivalis* infection. **(A)** Flow cytometry analysis showed the proportion of Ly6C^+^/CD31^+^ cells in the ZA-treated groups (ZA+/*P.g*- and ZA+/*P.g*+ groups) was lower than that in the ZA-untreated groups (ZA-/*P.g*- and ZA-/*P.g*+ groups) at 4 and 8 weeks postoperatively. While there was no statistical difference in the proportion of Ly6C+/CD31+ cells between the *P. gingivalis*-treated (ZA-/*P.g*+ and ZA+/*P.g*+) and *P. gingivalis*-untreated (ZA-/*P.g*- and ZA+/*P.g*-) groups at 4 and 8 weeks postoperatively. **(B)** TRAP staining showed the number of TRAP+ multinucleated cells in the ZA-treated groups was significantly lower than that of the ZA-untreated groups at 4 and 8 weeks postoperatively. While there was no statistical difference in the number of TRAP+ multinucleated cells between the *P. gingivalis*-treated and *P. gingivalis*-untreated groups at 4 and 8 weeks postoperatively. **(C)** Coomassie Brilliant Blue staining showed bone resorption area in the ZA-treated groups was smaller than that in the ZA-untreated groups at 4 and 8 weeks postoperatively. But no statistical difference was found in the bone resorption area between the *P. gingivalis*-treated and *P. gingivalis*-untreated groups at 4 and 8 weeks postoperatively. Bar: 50μm, 20× magnification. PO4W, postoperative 4 weeks; PO8W, postoperative 8 weeks. *p<0.05, **p<0.01.

Flow cytometry analysis showed, compared with the ZA-untreated groups (ZA-/*P.g*- and ZA-/*P.g*+ groups), the ZA-treated groups (ZA+/*P.g*- and ZA+/*P.g*+ groups) generated a smaller proportion of Ly6c^+^/CD31^+^ OCPs at both four and eight weeks after surgery (**Figure 4A**). TRAP staining showed a significantly lower number of TRAP positive osteoclasts in the ZA-treated groups compared to the ZA-untreated groups. This was found at both time points after surgery (**Figure 4B**). Bone resorption by osteoclasts derived from the ZA-treated groups at both four and eight weeks postoperatively was less and the resorption pits were smaller than those from the ZA-untreated groups (**Figure 4C**). These results indicated ZA administration suppressed osteoclastogenesis and bone resorption capacity.

There were no significant differences in the number of OCPs or osteoclasts and their bone resorption capacity between the *P. gingivali*s-treated (ZA-/*P.g*+ and ZA+/*P.g*+) and *P. gingivali*s-untreated (ZA-/*P.g*- and ZA+/*P.g*-) groups (**Figures 4A–C**). These findings showed that local *P. gingivali*s infection had no obvious effect on systemic osteoclastogenesis.

### ZA Administration and *P. gingivalis* Infection Altered the Expression Level of Osteoclastogenesis- and Inflammation-Related Genes

**Figure 5:**
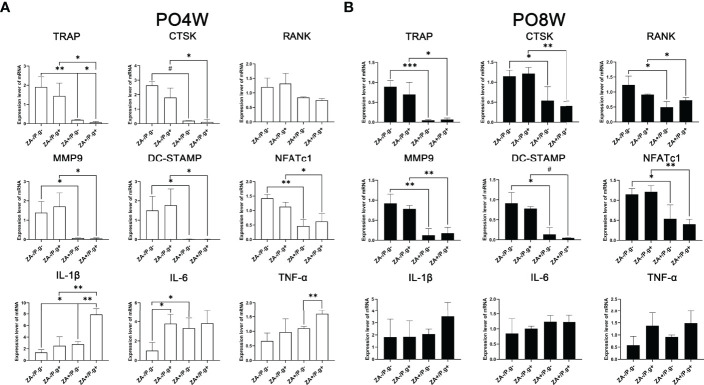
ZA administration and *P. gingivalis* infection altered the expression level of osteoclastogenesis- and inflammation-related genes. **(A)** PCR results of 4 weeks postoperatively. The expression level of osteoclastogenesis-related genes (TRAP, CTSK, MMP9, DC-Stamp and NFATc1) were significantly downregulated, the expression levels of inflammation-related genes (IL-1β and IL-6) were significantly upregulated in the ZA-treated groups compared to the ZA-untreated groups. There was no statistical difference in the expression level of osteoclastogenesis-related genes between the *P. gingivalis*-treated and *P. gingivalis*-untreated groups, while elevated expression levels of IL-1β, IL-6 and TNF-α were found in the *P. gingivalis*-treated groups. **(B)** PCR results of 8 weeks postoperatively. The expression level of osteoclastogenesis-related genes (TRAP, RANK, CTSK, MMP9, DC-Stamp and NFATc1) were significantly downregulated in the ZA-treated groups compared to the ZA-untreated groups, and there was no statistical difference in the expression levels of inflammation-related genes. No statistical difference was found in the expression level of osteoclastogenesis- and inflammation-related genes between *P. gingivalis*-treated and *P. gingivalis*-untreated groups. The values shown are normalized to GAPDH levels. The data represent mean ± SD of triplicate samples. PO4W, postoperative 4 weeks; PO8W, postoperative 8 weeks. *p<0.05, **p<0.01, ***p<0.001, ^#^*p*<0.0001.

The expression level of osteoclastogenesis-related genes including TRAP, CTSK, MMP9, DC-Stamp and NFATc1 were significantly downregulated in the ZA-treated groups compared to the ZA-untreated groups. This effect was found at both four and eight weeks after surgery (**Figures 5A, B**). These findings appeared to correlate with the inhibited effect of ZA on the osteoclastogenesis of OCPs. While *P. gingivali*s infection had no significant effect on the expression level of osteoclastogenesis-related genes (**Figures 5A, B**).

At four weeks postoperatively, the expression levels of inflammation-related genes including IL-1β and IL-6 were significantly upregulated in the ZA-treated groups, which coincided with the stimulation of ZA on cytokine production (**Figure 5A**). Besides, the *P. gingivalis*-treated groups also showed elevated expression levels of inflammation-related genes (**Figure 5A**). However, at the eighth week time point no statistical difference was found in the expression levels of inflammation-related genes among groups (**Figure 5B**).

### ZA Administration Inhibited the Expression Level of Osteoclastogenesis-Related Proteins Through P38 MAPK, ERK MAPK and P65 NF-κB Pathways

**Figure 6:**
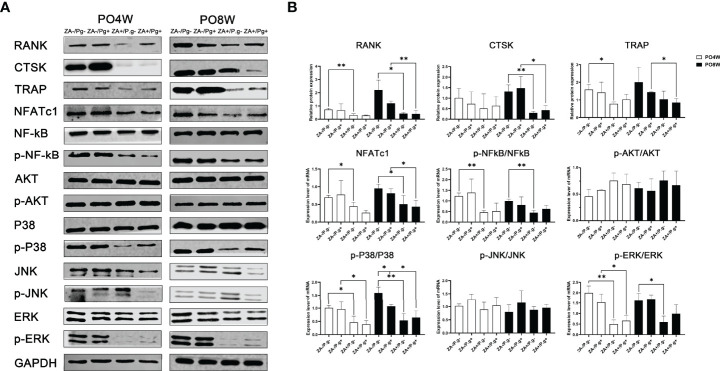
ZA administration inhibited the expression level of osteoclastogenesis-related proteins through P38 MAPK, ERK MAPK and P65 NF-κB pathways. **(A)** Protein extracts were harvested from the cultured cells and subjected to Western blot analysis. **(B)** Quantitative analysis showed the expression levels of RANK, TRAP and NFATc1 were decreased in osteoclasts derived from the ZA-treated groups at 4 or 8 weeks postoperatively. The expression level of CTSK in the ZA-treated groups was significantly lower than that of the ZA-untreated groups; an effect seen only at 8 weeks postoperatively. The results showed ZA administration significantly decreased phosphorylation of p38 MAPK, ERK MAPK, and p65 NF-kB molecules but had no effect on JNK MAPK and AKT at 4 or 8 weeks postoperatively. There was no statistical difference in the expression level of osteoclastogenesis- and pathways- related proteins between *P. gingivalis*-treated and *P. gingivalis*-untreated groups except for the p38 pathway at 8 weeks postoperatively. PO4W, postoperative 4 weeks; PO8W, postoperative 8 weeks. *p<0.05, **p<0.01.

Western blot assay was utilized to examine protein expression levels of a series of osteoclast differentiation markers, including CTSK, RANK, TRAP and NFATc1. The results showed the expression levels of RANK, TRAP and NFATc1 were all decreased in OCs derived from the ZA-treated groups compared to the ZA-untreated groups. This effect was seen at both four and eight weeks after surgery (**Figures 6A, B**). The expression level of CTSK in the ZA-treated groups was declined compared to the ZA-untreated groups at the eighth week time point; no statistical difference was found at four weeks after surgery (**Figure 6B**).

The MAPK and NF-κB, as well as AKT pathways, are critical in the regulation of osteoclastogenesis. Hence, we next evaluated the effect of ZA administration on these pathways. Western blot analyses indicated that ZA administration significantly decreased phosphorylation of p38, ERK, and p65 NF-kB but had no effect on JNK and AKT at both four and eight weeks after surgery, which might suggest ZA administration suppressed osteoclastogenesis *via* p38 MAPK, ERK MAPK and p65 NF-κB pathways (**Figures 6A, B**). Local *P. gingivalis* infection had no obvious effect on expression of osteoclastogenesis- and pathways- related proteins except for the p38 pathway at eight weeks after surgery (**Figures 6A, B**).

## Discussion

One of the most significant characteristics of BRONJ is its site-specific effect, but the underlying mechanism involved is still not entirely clear. Previous studies have suggested several possible explanations, such as (i) jaws have a stronger bisphosphonate intake capacity, (ii) jaws have a richer blood supply and a faster bone turnover rate than other bones (Marx et al., 2005; Wen et al., 2011); (iii) jaws are constantly subjected to mastication stress, resulting in the accumulation of microdamage; and (iv) some invasive dental treatments such as tooth extraction or placement of dental implants may cause BRONJ (Soma et al., 2021). Besides, the oral cavity contains a high level of bacterial flora and is susceptible to bacterial invasion especially after an intrusive dental operation, while bones in other parts of the body are in a closed environment and rarely exposed to bacteria (Kobayashi et al., 2010; Hinson et al., 2014; Rasmusson and Abtahi, 2014). Studies also have indicated that BPs could increase the bacterial load during early bone infection by decreasing lymphatic drainage and preventing the removal of necrotic bone that harbors bacteria (Li et al., 2010). Until now, the effect of bacteria on BRONJ has been widely discussed but the specific mechanism has not been well-established. In our study, we established a mouse model of bisphosphonate-related osteonecrosis in both femurs and jaws, and revealed the role of *P. gingivalis* in the occurrence of bisphosphonate-related osteonecrosis.

Some animal models have been introduced to demonstrate the association between bacterial infection and BRONJ *in vivo*. Bone exposure, lack of socket healing and osteonecrosis were present in jaws of most ZA-treated animals after extraction of teeth and bacterial presence was noted in areas of osteonecrotic alveolar bone (de Molon et al., 2014; Kuroshima et al., 2018b; Hadaya et al., 2019). In our study, we artificially put the femur in an environment that had direct contact with *P. gingivalis* and observed the healing of defects (Okumura et al., 2019). We found after administration of ZA, the femurs locally injected with *P. gingivalis* as well as the mandibles showed poor healing of wounds, with reduced bone volume and collagen fiber regeneration, increased empty lacunae and inflammatory infiltration, and declined active osteoclasts in the affected areas. In contrast, the ZA-untreated mandibles and the femurs showed rare necrotic bone and good bone healing. All above suggested, under ZA administration, local *P. gingivalis* application to the defected areas caused osteonecrosis of femurs. These findings indicate that ZA treatment in combination with the presence of oral bacteria can induce osteonecrosis in other bones of the body.

We also examined systemic effects of ZA treatment on osteoclastogenesis, and found its suppression; a finding consistent with previous studies (Ebetino et al., 2011; Kimachi et al., 2011; Russell, 2011). So it appears that ZA not only inhibits bone resorption by promoting apoptosis of mature osteoclasts (Hughes et al., 1995), the drug also negatively affects their formation. A possible explanation for this decreased level of osteoclast formation may be caused by a ZA-induced change in the precursors. Some studies suggested that nitrogen-containing BPs prevent OCPs from differentiating and migrating to osteolysis lesions (Kimachi et al., 2011; Nakagawa et al., 2020). We now demonstrated that ZA inhibited osteoclastogenesis by influencing the proportion of certain OCPs subtypes. De Vries and coworkers showed that osteoclasts were formed exclusively from bone marrow populations that contain four types of myeloid cells, of which myeloblasts (CD31^+^/Ly-6C^+^) were shown to develop into osteoclasts within a relatively short period of time (de Vries et al., 2009). According to our results, the percentage of these myeloblasts was lower in the ZA-treated groups compared to ZA-untreated groups. These findings suggest that ZA treatment inhibited osteoclastogenesis by reducing the proportion of myeloblasts. Moreover, we showed that ZA downregulated the NF-κB, p38 MAPKs and ERK MAPKs signaling pathways. These signaling pathways are downstream pathways of RANKL/RANK/TRAF6 signaling. The RANKL-RANK interaction sequentially results in the phosphorylation of NF-κB and MAPKs pathways, and promotes the activation of c-Fos and NFATc1. The stimulated NFATc1 translocates to the nucleus and activates expression of osteoclast marker genes, including RANK, CTSK, TRAP, and DC-STAMP (Tai et al., 2014; Kong et al., 2015; Xu et al., 2016; Dong et al., 2018; Huang et al., 2019). So it appears that ZA suppresses activation of NFATc1, and thus inhibits expression of downstream osteoclast marker genes, leading to inhibition of osteoclast differentiation and finally bone resorption. In addition, our results showed local *P. gingivalis* infection did not have a significant effect on systemic osteoclastogenesis, but only upregulated the expression level of some inflammation-related genes. That might be because systemic osteoclastogenesis is a complex process determined by numerous factors, and local stimulation might not cause system-level changes (Tsukasaki et al., 2020).

BRONJ is usually considered to differ from osteomyelitis in pathology and pathogenesis (De Antoni et al., 2018; Shuster et al., 2019; Wehrhan et al., 2019). Jaw osteomyelitis is a pathogen-induced infection of the bone marrow space, which occurs endogenously (hematogenous scattering—usually monomicrobial) or exogenously (trauma or iatrogenic effects—mostly polymicrobial). Osteocyte death in jaw bones and upregulated levels of inflammatory cytokines are required for BRONJ development (Rasmusson and Abtahi, 2014). Studies have shown that either ZA or *P. gingivalis* treatment elevated expression of inflammatory cytokines in OCPs. Thus, combining osteoclast inhibition with local inflammation due to infection may stimulate local inflammatory cytokine levels beyond levels that osteocytes can endure, promoting apoptosis and resulting in BRONJ development (Lesclous et al., 2009). We observed that treatment with ZA inhibited osteoclastogenesis but increased levels of inflammatory cytokines both *in vivo* and *in vitro*. We assume that under the influence of ZA, marrow cells transform into macrophages that secrete inflammatory factors and suppress the differentiation into osteoclasts.

The sequence of events resulting in osteonecrosis is still controversial. Some studies mentioned the appearance of necrotic bone may precede oral bacterial infections (Nicolatou-Galitis et al., 2015). Local high bone concentrations of ZA would be toxic for adjacent soft tissue, resulting in mucosal disruption and delayed healing, thus facilitating secondary colonization of the necrotic bone by the oral flora. The data of our study suggested the osteonecrosis of femur occurred only when ZA administration and local bacterial irritation were given at the same time. This strongly suggest bacteria is one of the initiating factors of BRONJ. They first colonize the bone defects and then lead to local infection and necrosis. Some studies referred to such an “outside-in” process in which mucosal damage provides oral bacteria with access to the underlying bone, leading to bone infection and necrosis (Reid et al., 2007; Landesberg et al., 2008; Scheper et al., 2009).

According to some clinical cases, periodontitis may predispose patients to developing BRONJ (Marx et al., 2005; Badros et al., 2006). It was noted in a seminal paper that 84% of BRONJ patients had periodontal disease, including 29% with advanced disease (Marx et al., 2005). The American Association of Oral and Maxillofacial Surgeons (AAOMS) has reported that individuals with periodontal disease have a 7-fold greater risk of developing osteonecrosis of the jaw (AAOMS, 2007). Apart from periodontitis, the dosage, type and administration method of BPs are also important risk factors for BRONJ (Saad et al., 2012; Fliefel et al., 2015). According to some studies, the oncological dosage of intravenous BPs was related to a greater risk of BRONJ when compared to dosing schemes used in osteoporosis treatment (Fliefel et al., 2015; von Moos et al., 2019). Patients who were given additional antiangiogenic and immunosuppressive medications were more often affected with BRONJ. The above reminds us that in clinical practice, it is necessary to improve oral hygiene for patients who are about to receive BPs treatment. At the same time, attention should also be paid to the dosage and administration methods of BPs to prevent BRONJ.

In conclusion, we established a mouse model of bisphosphonate-related osteonecrosis in both femur and jaw. Our findings suggest that the site specificity of bisphosphonate-related osteonecrosis is associated with the presence of (oral) bacteria. Moreover, our findings contribute to a further elucidation of the underlying pathogenesis of BRONJ: inhibition of osteoclastogenesis by decreasing the number of precursors and affecting intracellular pathways essential for the formation of osteoclasts.

## Data Availability Statement

The original contributions presented in the study are included in the article/**Supplementary Material**, further inquiries can be directed to the corresponding author.

## Ethics Statement

The animal study was reviewed and approved by Institutional Animal Care and Use Committee approval of Wuhan University.

## Author Contributions

QW and SW conceived this investigation and designed all experiments. SW, FL, JT, XY, YL, and NL performed the included experiments. QW, SW, and VE contributed to data analysis and manuscript drafting. QW, SW, and VE contributed to manuscript revision. All authors read and approved the submitted version.

## Funding

This study was supported by grants from the National Natural Science Foundation of Hubei Province of China (General Program, 2020CFB841).

## Conflict of Interest

The authors declare that the research was conducted in the absence of any commercial or financial relationships that could be construed as a potential conflict of interest.

## Publisher’s Note

All claims expressed in this article are solely those of the authors and do not necessarily represent those of their affiliated organizations, or those of the publisher, the editors and the reviewers. Any product that may be evaluated in this article, or claim that may be made by its manufacturer, is not guaranteed or endorsed by the publisher.
